# Developing a *Bacteroides* System for Function-Based Screening of DNA from the Human Gut Microbiome

**DOI:** 10.1128/mSystems.00195-17

**Published:** 2018-03-27

**Authors:** Kathy N. Lam, Eric C. Martens, Trevor C. Charles

**Affiliations:** a Department of Biology, University of Waterloo, Waterloo, Canada; b Department of Microbiology and Immunology, University of Michigan, Ann Arbor, Michigan, USA; Dalhousie University

**Keywords:** Bacteroides thetaiotaomicron, anaerobic sulfatase maturating enzyme, chondroitin sulfate utilization, fosmid library, functional metagenomics, functional screening, gut microbiota, human gut microbiome, metagenomic library, surrogate host

## Abstract

Functional metagenomics is a powerful method that allows the isolation of genes whose role may not have been predicted from DNA sequence. In this approach, first, environmental DNA is cloned to generate metagenomic libraries that are maintained in Escherichia coli, and second, the cloned DNA is screened for activities of interest. Typically, functional screens are carried out using E. coli as a surrogate host, although there likely exist barriers to gene expression, such as lack of recognition of native promoters. Here, we describe efforts to develop Bacteroides thetaiotaomicron as a surrogate host for screening metagenomic DNA from the human gut. We construct a B. thetaiotaomicron-compatible fosmid cloning vector, generate a fosmid clone library using DNA from the human gut, and show successful functional complementation of a B. thetaiotaomicron glycan utilization mutant. Though we were unable to retrieve the physical fosmid after complementation, we used genome sequencing to identify the complementing genes derived from the human gut microbiome. Our results demonstrate that the use of B. thetaiotaomicron to express metagenomic DNA is promising, but they also exemplify the challenges that can be encountered in the development of new surrogate hosts for functional screening.

**IMPORTANCE** Human gut microbiome research has been supported by advances in DNA sequencing that make it possible to obtain gigabases of sequence data from metagenomes but is limited by a lack of knowledge of gene function that leads to incomplete annotation of these data sets. There is a need for the development of methods that can provide experimental data regarding microbial gene function. Functional metagenomics is one such method, but functional screens are often carried out using hosts that may not be able to express the bulk of the environmental DNA being screened. We expand the range of current screening hosts and demonstrate that human gut-derived metagenomic libraries can be introduced into the gut microbe Bacteroides thetaiotaomicron to identify genes based on activity screening. Our results support the continuing development of genetically tractable systems to obtain information about gene function.

## Abstract (precis)

Human gut microbiome research has been supported by advances in DNA sequencing that make it possible to obtain gigabases of sequence data from metagenomes but is limited by a lack of knowledge of gene function that leads to incomplete annotation of these data sets. There is a need for the development of methods that can provide experimental data regarding microbial gene function. Functional metagenomics is one such method, but functional screens are often carried out using hosts that may not be able to express the bulk of the environmental DNA being screened. We expand the range of current screening hosts and demonstrate that human gut-derived metagenomic libraries can be introduced into the gut microbe Bacteroides thetaiotaomicron to identify genes based on activity screening. Our results support the continuing development of genetically tractable systems to obtain information about gene function.

## INTRODUCTION

**FIG 1:**
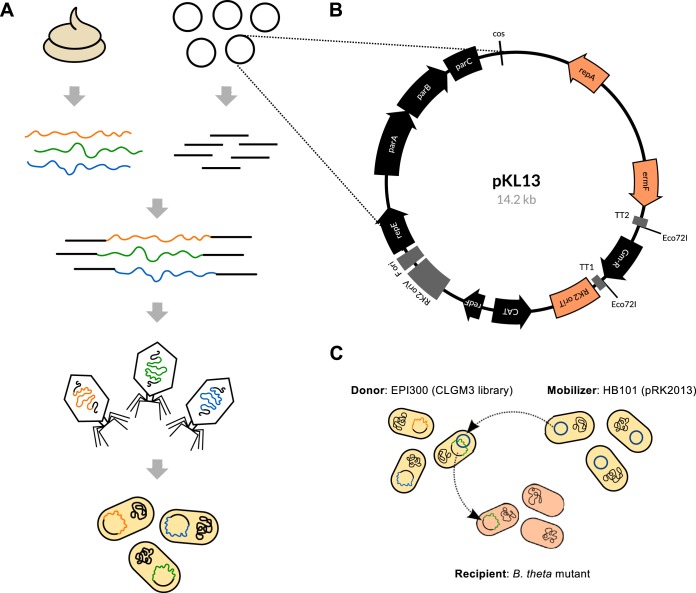
Overview of the functional metagenomics approach and development of a B. thetaiotaomicron-compatible system to screen gut-derived DNA. (A) Metagenomic library construction using DNA from the human gut: high-molecular-weight DNA is isolated from feces and ligated to a vector with a *cos* site, allowing lambda phage packaging and transduction of E. coli to generate the library. Clones comprising the metagenomic library are typically pooled and saved as frozen stocks for future screening. Figure is adapted from reference 10. (B) Vector map of pKL13, a mobilizable B. thetaiotaomicron-compatible fosmid vector. Map generated using AngularPlasmid. (C) Depiction of the triparental conjugation to transfer a metagenomic library from E. coli to a B. thetaiotaomicron recipient for functional complementation of a mutant trait. The pKL13 fosmid is not self-mobilizable. The helper plasmid pRK2013, expressing *tra* genes, is transferred from the mobilizer strain to the donor strain, allowing the subsequent transfer of the library from the donor strain to the recipient strain. B. theta, B. thetaiotaomicron.

As the microbes that live within the human body are implicated in a growing number of human disease states, there has been corresponding growing interest in the role of the different organisms that comprise the gut microbiota. This interest has been supported by advances in DNA sequencing technology that allow the generation of large metagenome sequence data sets, and yet, study of the human gut microbiota is hampered by a lack of knowledge of gene function that makes annotation of those data sets incomplete; for example, approximately 50% of genes identified in Human Microbiome Project (HMP) stool samples lacked a functional assignment using standard (GO, EC, or homology-based) annotation methods (1). As research on the microbiota continues, there will be an increased need for effective methods that can provide knowledge of microbial gene function (2). Functional metagenomics is an approach in which environmental DNA is cloned to generate metagenomic libraries that are maintained in Escherichia coli (Fig. 1A) and the cloned DNA is screened en masse for specific functions of interest. This powerful approach allows the isolation of genes whose roles may not have been predicted based on their DNA sequence alone (3), but crucially, the method is dependent on the ability to express cloned metagenomic DNA in a surrogate host.

The human gut microbiota is dominated by members of the *Firmicutes* and *Bacteroidetes* phyla, with the *Bacteroides* genus often the most abundant (4). Though E. coli, a member of the *Gammaproteobacteria*, is often used as a surrogate host to screen gut-derived DNA, there is evidence of a barrier to the expression of *Bacteroides* genes at the level of transcription (5), due to differences in promoter consensus recognition between the *Bacteroidetes* primary sigma factor and that of E. coli (6). The development of a more suitable host will likely improve hit rates from activity screens (7), and in particular, a *Bacteroides* host would offer an array of potentially selectable glycan utilization phenotypes (8, 9) that are less feasible in E. coli. The human gut symbiont Bacteroides thetaiotaomicron is a natural choice as a surrogate host to screen gut metagenomic DNA, given that molecular genetic methods for this organism are reasonably well developed. Here, we describe our attempt to develop B. thetaiotaomicron for functional metagenomics, through the construction of a B. thetaiotaomicron-compatible fosmid cloning vector, generation of a human gut metagenomic library, and screening of the library to achieve functional complementation of a B. thetaiotaomicron glycan utilization mutant.

## RESULTS

### Construction of a *Bacteroides*-compatible human gut metagenomic library.

To be able to screen a library in a B. thetaiotaomicron host, the library must be constructed using a vector that is able to replicate in B. thetaiotaomicron. To construct a suitable library cloning vector, we chose to build on pCC1FOS, a commercial fosmid vector that has been widely used for constructing metagenomic libraries from diverse environments (10). Although cosmid vectors for B. thetaiotaomicron have been constructed in the past using pBR322 and RSF1010 origins (11, 12), we desired the potentially increased insert stability offered by a single-copy F-based vector, as cloned *Bacteroides* DNA may be unstable in E. coli (12) and instability may be exacerbated by maintenance at a higher copy number (13). We chose to use a self-replicating rather than an integrating vector because the former allows fosmid DNA to be isolated from B. thetaiotaomicron cells by plasmid minipreparation, facilitating DNA sequencing of the complementing insert. A fosmid vector was especially desirable for two reasons: (i) lambda packaging to generate fosmid clone libraries is very efficient and (ii) large-fragment libraries would be suitable for capturing the polysaccharide utilization loci of *Bacteroides*, which may contain over 20 genes (9).

pCC1FOS was modified by the addition of an origin of transfer (*oriT*) to allow plasmid conjugation from E. coli to B. thetaiotaomicron, as well as plasmid replication elements (*repA*) and an erythromycin-selectable marker (*ermF*) for B. thetaiotaomicron. The constructed B. thetaiotaomicron-compatible vector was designated pKL13 (Fig. 1B) and used to generate a human gut metagenomic library, called CLGM3, that contained over 100,000 unique clones with an estimated average insert size of 26 ± 10 kb. To assess the level of transfer from E. coli to B. thetaiotaomicron, a triparental conjugation was carried out (Fig. 1C), resulting in conjugation efficiencies (relative to recipient) of 2.6 × 10^−2^ for the vector alone and 1.1 × 10^−2^ for the CLGM3 metagenomic library. Though transfer into B. thetaiotaomicron was not as efficient as that for other surrogate hosts, such as the legume symbiont Sinorhizobium meliloti (14), it was sufficient for initial attempts at functional complementation.

### Proof-of-principle functional complementation of a *B. thetaiotaomicron anSME* mutant.

**FIG 2:**
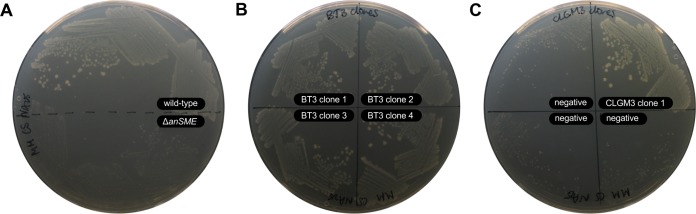
Functional complementation of B. thetaiotaomicron *anSME* mutant on chondroitin sulfate as sole carbon source. (A) Comparison of B. thetaiotaomicron VPI 5482 wild-type and Δ*anSME* phenotypes on chondroitin sulfate as sole carbon source. (B and C) Streak-purified complementing clones in the *anSME* background, isolated from a B. thetaiotaomicron genomic library (BT3) and a human gut metagenomic library (CLGM3), respectively.

As a host for a proof-of-principle functional complementation, we chose a B. thetaiotaomicron Δ*anSME* mutant, also called the Δ*chuR* mutant (15). The 1,245-bp *chuR*/*anSME* gene (BT_0238) was first identified through transposon mutagenesis as a *r*egulator of *ch*ondroitin sulfate and heparin *u*tilization (16). Knocking out this gene renders B. thetaiotaomicron unable to grow on the glycan chondroitin sulfate or heparin as a sole carbon source. It was later characterized as an *an*aerobic *s*ulfatase *m*aturating *e*nzyme: the breakdown of these glycans by B. thetaiotaomicron requires the action of sulfatase enzymes that must be modified posttranslationally by the product of the *anSME* gene (17); without this modification, the sulfatases are inactive. The mutant phenotype being dependent on the single *anSME* gene, as well as the clean phenotype of the Δ*anSME* mutant on chondroitin sulfate (Fig. 2A), made it a good candidate for functional complementation.

To screen the CLGM3 metagenomic library for genes able to complement the *anSME* mutant, the library was conjugated from E. coli EPI300 into the B. thetaiotaomicron Δ*anSME* strain, selecting on minimal medium with chondroitin sulfate as the sole carbon source. As a positive control, a B. thetaiotaomicron genomic library (constructed using B. thetaiotaomicron VPI 5482 DNA; called BT3) was screened simultaneously. Colonies arising on the selective medium were streak purified to confirm the restored phenotype, providing evidence that the mutant’s ability to grow on chondroitin sulfate was restored by complementation with clones from the B. thetaiotaomicron genomic library or the gut metagenomic library (Fig. 2B or C, respectively).

### Possible fosmid clone recombination into the *B. thetaiotaomicron* host genome.

Plasmid DNA can be prepared from B. thetaiotaomicron cultures using standard alkaline lysis, and we confirmed that plasmid preparations of empty vector DNA from B. thetaiotaomicron can be used to successfully transform E. coli EPI300. We applied this same strategy to obtain the fosmid DNA from cultures of the complemented *anSME* mutant; however, plasmid minipreparations followed by transformation of EPI300 yielded no transformants for the samples, indicating that there was no fosmid DNA isolated from these cultures despite the restored ability to use chondroitin sulfate as the sole carbon source. We hypothesized that the *anSME*-complementing fosmid DNA may have recombined into the host genome, an unfortunate scenario as the screening of pooled-clone metagenomic libraries hinges on being able to retrieve the complementing DNA for sequence analysis.

**FIG 3:**
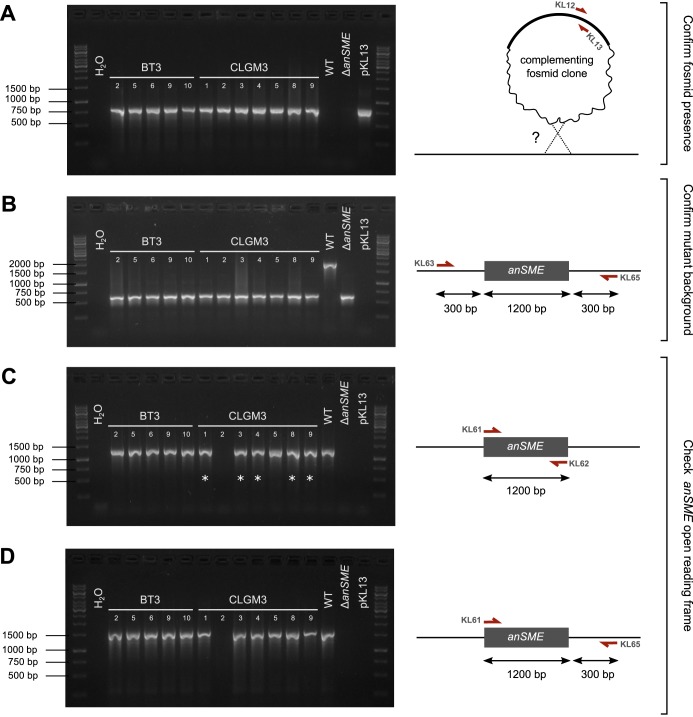
PCR analysis of genomic DNA isolated from *anSME*-complemented B. thetaiotaomicron clones. As controls, genomic DNAs from the wild-type (WT) B. thetaiotaomicron VPI 5482 and the Δ*anSME* mutant were included, as well as plasmid DNA for the pKL13 fosmid. PCR was carried out to amplify the *oriT* sequence of the fosmid vector backbone (~800 bp) (A), a product corresponding to 300 bp upstream and 300 bp downstream of the *anSME* ORF (~1,800 bp for VPI 5482 and 600 bp for Δ*anSME*) (B), the *anSME* ORF (~1,200 bp for VPI 5482) (asterisks indicate products confirmed as identical to B. thetaiotaomicron VPI 5482 by Sanger sequencing) (C), and the *anSME* ORF plus 300 bp downstream (~1,500 bp for VPI 5482) (D).

To test this hypothesis, we isolated genomic DNA from 5 clones from the BT3 complementation and 7 clones from the CLGM3 complementation and used the DNA as the template in a PCR to test for the presence of the fosmid’s *oriT* sequence. As suspected, the genomic DNA preparations from all clones were positive for the *oriT* (Fig. 3A). We also confirmed the *anSME* mutant background; this mutant strain carries a deletion of the ~1,200-bp *anSME* open reading frame (ORF), and primers designed to 300 bp upstream and 300 bp downstream of the ORF amplify only 600 bp from the mutant versus ~1,800 bp from the wild type. As expected, genomic DNA preparations from all of the BT3- and CLGM3-complemented clones carried the mutant *anSME* genomic context (Fig. 3B).

We next asked whether the metagenomic fosmid clones were carrying DNA from B. thetaiotaomicron or closely related species, which may explain the propensity for homologous recombination. To answer this, we performed PCR for the *anSME* ORF using primers based on the B. thetaiotaomicron VPI 5482 *anSME* sequence, which would likely amplify only exact or close matches to B. thetaiotaomicron. All clones from the B. thetaiotaomicron BT3 library produced PCR products (Fig. 3C), which was expected as this library was constructed using B. thetaiotaomicron DNA. From the CLGM3 metagenomic library, all but clone 2 showed amplification, confirming our suspicion that most complementing clones were probably closely related to the host. We purified and Sanger sequenced the PCR products, finding that 5 of the 6 metagenomic *anSME* sequences were an exact match to VPI 5482. The last of the 6 products, from CLGM3 clone 5, was not identical but highly similar (see Fig. 5).

The result of the PCR for the *anSME* ORF and flanking region (Fig. 3B) was surprising in that the BT3 library clones did not exhibit both the 600-bp and 1,800-bp bands—the former from the B. thetaiotaomicron Δ*anSME* background and the latter from the complementing fosmid DNA carrying the B. thetaiotaomicron *anSME* gene. To determine if the smaller product may be preferentially amplified in PCR, we designed primers to the *anSME* locus such that the smaller PCR product was not possible, amplifying the *anSME* ORF plus 300 bp downstream (Fig. 3D). The ~1,500-bp product of this PCR confirmed that indeed the complementing *anSME* locus was present in the clones carrying VPI 5482 DNA (BT3 clones) or closely related metagenomic DNA (CLGM3 clones).

### Genome sequencing of *B. thetaiotaomicron* Δ*anSME* complemented with metagenomic DNA.

We were interested in further characterizing CLGM3 clone 2 and clone 5; these derived from the metagenomic library and appeared to be carrying DNA distinct from VPI 5482. We decided to carry out genome sequencing in the hope that we could (i) gain insight into the sequence similarity between fosmid clone and host that may contribute to recombination and (ii) identify the complementing *anSME* gene from clone 2, which we were unable to retrieve using PCR (Fig. 3C). After sequencing, we first aligned reads to the B. thetaiotaomicron VPI 5482 genome; second, we *de novo* assembled reads to identify pKL13 vector DNA and the complementing *anSME* genes.

**FIG 4:**
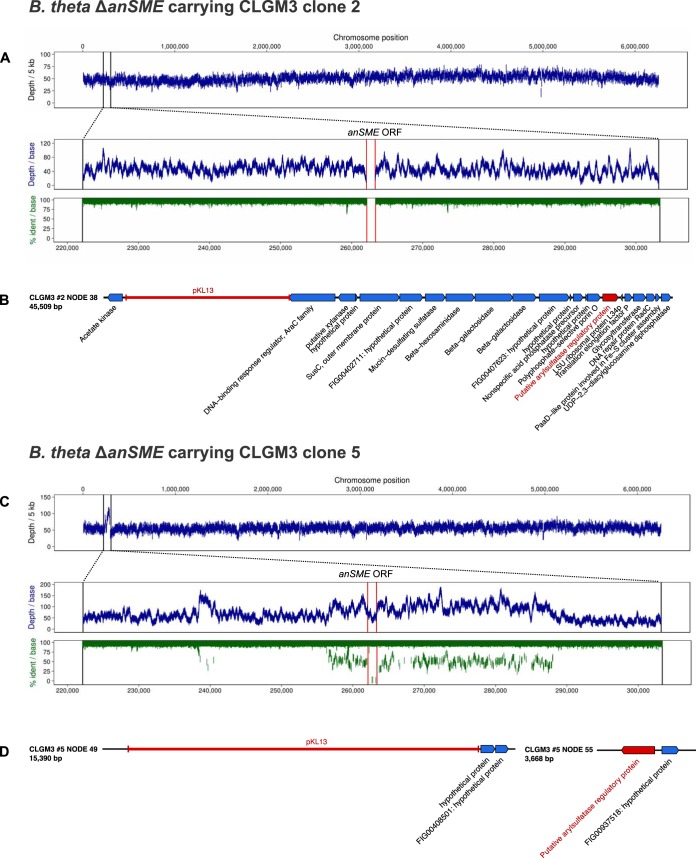
Genome sequencing and *de novo* assembly results for B. thetaiotaomicron Δ*anSME* carrying CLGM3 clone 2 and clone 5. (A and C) Mean read depth per 5,000 bp after mapping reads to VPI 5482 genome. (Pullout) Read depth and percent identity per base pair at the *anSME* locus; red lines delineate *anSME* open reading frame. (B and D) Relevant contigs from *de novo* assembly; pKL13 vector sequence and complementing *anSME* gene are indicated in red. B. theta, B. thetaiotaomicron.

By mapping reads from clone 2 back to VPI 5482, we were able to confirm the Δ*anSME* mutant background through the zero read depth observed at the cleanly deleted *anSME* ORF (Fig. 4A). Assembly of reads resulted in a fosmid-sized 45-kb contig that included the pKL13 vector backbone and the complementing *anSME* gene (Fig. 4B). The insert carried by the fosmid had 99% nucleotide identity to Bacteroides vulgatus ATCC 8482 and was sufficiently different from VPI 5482 to enable essentially complete assembly; this dissimilarity also validates the lack of an amplicon from PCR using primers that were designed against the VPI 5482 *anSME* sequence (Fig. 3C and D). Interestingly, the ends of this contig share sequence similarity with regions that flank a gene annotated as a transposase in the host genome, suggesting a possible integration mechanism and/or locus, although there exist other regions along the contig of lower similarity to the host genome (see Fig. S1 in the supplemental material).

10.1128/mSystems.00195-17.1 FIG S1  Regions of sequence similarity between a contig derived from CLGM clone 2 (bottom) and the B. thetaiotaomicron host genome (top). B. theta, B. thetaiotaomicron. Download FIG S1, EPS file, 0.6 MB. Copyright © 2018 Lam et al. 2018 Lam et al. This content is distributed under the terms of the Creative Commons Attribution 4.0 International license.

When we mapped reads from clone 5 back to VPI 5482, we found that there were reads with high-enough similarity to map to the VPI 5482 *anSME* locus. However, the higher read depth at that locus as well as the low identity observed indicated a foreign source of DNA; perhaps most tellingly, the stretch of low identity was consistent with the size of a typical fosmid insert (Fig. 4C). The high sequence similarity between the fosmid insert and host genome likely also contributed to a fragmented *de novo* assembly: a fosmid-sized contig was not assembled, although the pKL13 vector and *anSME* sequence were found on smaller contigs (Fig. 4D). The sequences adjacent to the vector (on both ends) as well as the contig carrying the *anSME* gene exhibited high nucleotide identity to B. thetaiotaomicron VPI 5482 (84 to 99%). Due to the fragmented nature of assembly and the high similarity between metagenomic DNA and host DNA, it was difficult to speculate on possible integration loci for this clone.

### Gut-derived *anSME* genes identified by functional complementation.

**FIG 5:**
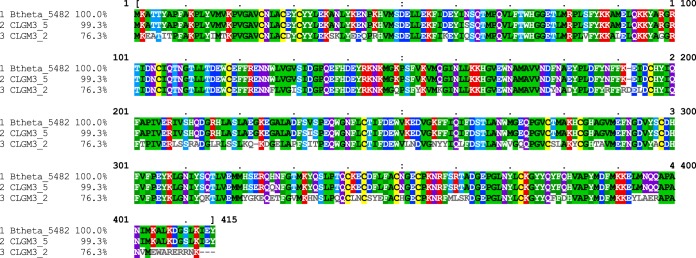
Multiple sequence alignment of the B. thetaiotaomicron VPI 5482 *anSME* gene and the metagenomic *anSME* genes from CLGM3 library clones 2 and 5. Translated nucleotide sequences were aligned to the B. thetaiotaomicron VPI 5482 protein sequence using MUSCLE version 3.8 (38) and visualized with MView (39). Percent identity is indicated on the left; residues differing from VPI 5482 are indicated in white. Btheta, B. thetaiotaomicron.

Using functional complementation, we were able to identify two *anSME* gene sequences from a gut metagenomic library (Fig. 4B and D). Comparison of the translated sequences to the VPI 5482 *anSME* 415-residue protein sequence revealed a number of changes at the amino acid level (Fig. 5), none of which were in the three conserved cysteine clusters thought to be involved in the ability of the *anSME* gene product to mature sulfatase enzymes (17).

The *anSME* gene of clone 2 was identical to B. vulgatus ATCC 8482, whereas the *anSME* gene of clone 5 appeared to be novel though nearly identical to B. thetaiotaomicron VPI 5482. The identification of *anSME* genes from a human gut metagenomic library that are different in sequence from the B. thetaiotaomicron VPI 5482 host used for screening indicates that functional screening of metagenomic libraries using B. thetaiotaomicron is a promising strategy, although the issue of possible clone recombination will need to be addressed.

## DISCUSSION

In this study, B. thetaiotaomicron was chosen as a host for screening gut-derived metagenomic DNA because it is anticipated to be able to express a greater fraction of the cloned DNA than would E. coli. Previous work on the B. thetaiotaomicron 16S rRNA gene operon showed that while the B. thetaiotaomicron ribosome-binding site was recognized by E. coli, the barrier to gene expression was due to lack of promoter recognition (5). Though there have been reports in the literature of functional screens in E. coli yielding positive clones carrying *Bacteroides*-derived DNA (18–22), these hits may be due to spurious transcription of foreign DNA in E. coli rather than transcription from native *Bacteroides* promoters.

To develop a system that may be better suited for screening gut metagenomic libraries, we generated B. thetaiotaomicron-compatible fosmid libraries with which we demonstrated successful functional complementation of a B. thetaiotaomicron *anSME* mutant. Analysis of the complemented mutants, however, indicated that recombination may have occurred between the host genome and the DNA carried on the fosmid clones, suggesting high sequence similarity between the host genome and complementing DNA. Consistent with this, we found that nearly all complementing *anSME* genes from the metagenomic library were exact or close matches to B. thetaiotaomicron, which may not be surprising given that B. thetaiotaomicron is often a dominant species in the human distal gut (23). We carried out full genome sequencing on 2 of the 7 metagenomic clones to obtain the complementing *anSME* genes and found both deriving from *Bacteroides* species. It is possible that recombination may be due to instability of the pKL13 vector backbone; however, we did confirm that empty vector could be introduced into and reisolated from B. thetaiotaomicron.

To demonstrate that B. thetaiotaomicron can be used as a screening host, we chose a Δ*anSME* mutant for the simplicity of its phenotype being dependent on a single gene. In retrospect, this choice may have limited the diversity of hits obtained. Although functional metagenomics is an approach that can uncover novel genes, here we found that the *anSME*-complementing genes obtained from the gut were either identical or closely related to B. thetaiotaomicron VPI 5482. To enrich for more diverse hits, it may be useful to choose a target with a larger known sequence space, including multigene operons; indeed, the strength of the functional metagenomic approach is that the large inserts of *cos*-based vectors can carry entire operons involved in polysaccharide utilization. Here, we showed that an ~45-kb fosmid carrying human gut metagenomic DNA can be successfully conjugated into B. thetaiotaomicron, demonstrating that large, multigene operons characteristic of polysaccharide utilization loci can be transferred for functional complementation.

Although the propensity for homologous recombination presents difficulties for the screening of pooled-clone metagenomic libraries, it is not a barrier to the functional metagenomics approach. One possible solution is to use arrayed libraries in which clones are stored and conjugated into the recipient individually, enabling clone tracking and eliminating the need for clone DNA retrieval. Individual conjugations may also be advantageous over en masse conjugations if the relatively low conjugation efficiency is a bottleneck for transferring large clone libraries into a B. thetaiotaomicron recipient. Screening of arrayed libraries containing hundreds of thousands of clones, however, often requires specialized equipment to achieve the necessary throughput. Another possible solution may be to use a recombination-deficient B. thetaiotaomicron strain, although a B. thetaiotaomicron *recA* mutant has been reported to have increased sensitivity to oxygen (24).

Our results show that the development of a B. thetaiotaomicron system for functional screening was not as straightforward as hoped; however, the genetic tractability of B. thetaiotaomicron and the generation of genetic tools with which to manipulate it (25) provide support for continued efforts. In addition, a more quantitative comparison between E. coli and B. thetaiotaomicron as expression hosts for metagenomic library screening would be valuable. Although the development of a B. thetaiotaomicron expression host may offer an advantage over E. coli for screening DNA derived from the *Bacteroidetes*, B. thetaiotaomicron would not be ideal for screening DNA from other phyla present in the gut, particularly *Firmicutes*. A comprehensive screening strategy will likely require the use of multiple expression hosts. Indeed, expanding the range of screening hosts will be important for the functional metagenomics field and for the characterization of microbial genes with currently unknown function. The fosmid vector that we have described in this report, and the strategy for functional complementation via conjugal transfer, will provide a strong foundation for further refinements.

## MATERIALS AND METHODS

### Bacterial strains, plasmids, and oligonucleotides.

The bacterial strains and plasmids used in this study are provided in Table 1. Oligonucleotide names and sequences are provided in Table 2.

**TABLE 1:** Bacterial strains and plasmids used in this study

Strain or plasmid	Description	Source or reference
Strains		
E. coli		
EPI300	F^−^ *mcrA Δ*(*mrr-hsdRMS-mcrBC*) φ80d*lacZ*ΔM15 Δ*lacX74 recA1 endA1 araD139* Δ(*ara leu*)*7697 galU galK rpsL* (Sm^r^) *nupG trfA dhfr*	Epicentre
HB101	F^−^ *mcrB mrr hsdS20*(r_B_^−^ m_B_^−^) *recA13 leuB6 ara-14 proA2 lacY1 galK2 xyl-5 mtl-1 rpsL20* (Sm^r^) *glnV44*	40
B. thetaiotaomicron		
VPI 5482	B. thetaiotaomicron type strain; the VPI 5482 type strain is the same as ATCC 29148	23
BtUW24	Derivative of VPI 5482 with deletion of BT_2275 (*tdk*); used in conjunction with pExchange-*tdk* to construct deletion mutants	30
BtUW25	Derivative of Δ*tdk* with deletion of BT_0238 (*anSME*); unable to grow on chondroitin sulfate as sole carbon source	15
BtUW4	B. thetaiotaomicron BtUW25 carrying *anSME*-complementing clone from BT3 genomic library designated BT3_chuR2	This study
BtUW7	B. thetaiotaomicron BtUW25 carrying *anSME*-complementing clone from BT3 genomic library designated BT3_chuR5	This study
BtUW8	B. thetaiotaomicron BtUW25 carrying *anSME*-complementing clone from BT3 genomic library designated BT3_chuR6	This study
BtUW11	B. thetaiotaomicron BtUW25 carrying *anSME*-complementing clone from BT3 genomic library designated BT3_chuR9	This study
BtUW12	B. thetaiotaomicron BtUW25 carrying *anSME*-complementing clone from BT3 genomic library designated BT3_chuR10	This study
BtUW14	B. thetaiotaomicron BtUW25 carrying *anSME*-complementing clone from CLGM3 metagenomic library designated CLGM3_chuR1	This study
BtUW15	B. thetaiotaomicron BtUW25 carrying *anSME*-complementing clone from CLGM3 metagenomic library designated CLGM3_chuR2 (clone 2)	This study
BtUW16	B. thetaiotaomicron BtUW25 carrying *anSME*-complementing clone from CLGM3 metagenomic library designated CLGM3_chuR3	This study
BtUW17	B. thetaiotaomicron BtUW25 carrying *anSME*-complementing clone from CLGM3 metagenomic library designated CLGM3_chuR4	This study
BtUW18	B. thetaiotaomicron BtUW25 carrying *anSME*-complementing clone from CLGM3 metagenomic library designated CLGM3_chuR5 (clone 5)	This study
BtUW20	B. thetaiotaomicron BtUW25 carrying *anSME*-complementing clone from CLGM3 metagenomic library designated CLGM3_chuR8	This study
BtUW21	B. thetaiotaomicron BtUW25 carrying *anSME*-complementing clone from CLGM3 metagenomic library designated CLGM3_chuR9	This study
Plasmids		
pRK2013	Mobilizer plasmid; ColE1 replication origin and Km^r^	41
pAFD1	E. coli-*Bacteroides* shuttle vector with pUC replication origin	42
pJC8	Cosmid vector with RK2 replication origin; NCBI accession no. KC149513	29
pJET1.2	Commercial vector for PCR product cloning; NCBI accession no. EF694056	43
pCC1FOS	Commercial fosmid vector; NCBI accession no. EU140751	Epicentre
pKL13	Derivative of pCC1FOS; *ermF* and *repA* for selection and replication in *Bacteroides*, respectively; *oriT* for conjugation from E. coli into B. thetaiotaomicron; NCBI accession no. KU746975	This study

**TABLE 2:** Oligonucleotides used in this study

Oligonucleotide	Purpose	Sequence
KL12	F primer to amplify RK2 *oriT* from pJC8, with HindIII adapter	CCTAAGCTTTCGGTCTTGCCTTGCTCGTCGG
KL13	R primer to amplify RK2 *oriT* from pJC8, with HindIII adapter	CCTAAGCTTGCGCTTTTCCGCTGCATAACCC
KL14	F primer to amplify *ermF-repA* fragment from pAFD1, with EcoRI adapter	CCTGAATTCACTTTTGTGCAATGTTGAAGATTAGTAATTCTATTC
KL15	R primer to amplify *ermF-repA* fragment from pAFD1, with EcoRI adapter	CCTGAATTCATAACAGCCGGTGACAGCCGGC
KL16	Primer walking *ermF-repA* fragment	GTTCAACCAAAGCTGTGTCGTTTTCAATAGC
KL33	Primer walking *ermF-repA* fragment	CAGGTATGCCAAACGTGGTTCTAAAAATGC
KL42	Primer walking *ermF-repA* fragment	GGAACTGCAAAATTCCTAAAATCACAACC
KL43	Primer walking *ermF-repA* fragment	CAAGCCCGTCAGGGCGCGTCAGCGGGTGTTGG
KL45	Primer walking *ermF-repA* fragment	AACAGACAAAGCCGTTTATAAAGGACTTGC
KL46	Primer walking *ermF-repA* fragment	GTCAGCAACAAAGGTAGTACTTTATTATCG
KL61	F primer for B. thetaiotaomicron *anSME* ORF (BT_0238)	ATGAAAGCAACAACTTATGCACCTTTTGCCAAACC
KL62	R primer for B. thetaiotaomicron *anSME* ORF (BT_0238)	TTAATATTCTATTTTTAAACTTCCGTCTTTTAGTGCTTTC
KL63	F primer for 300 bp upstream of B. thetaiotaomicron *anSME* ORF	TCTCCATCCCTCAAAGTCTTCAGATATAACATTTTTCC
KL65	R primer for 300 bp downstream of B. thetaiotaomicron *anSME* ORF	TAACCGCAGTGATGGTTAGTCAGGATCAAGC
KL67	Sequence *anSME* PCR product from clone CLGM3_chuR5	AAGCGGACGCATCAGCGTTTCTCCACC
KL69	Sequence *anSME* PCR product from clone CLGM3_chuR5	TCTATTTGCCTGCAACGGAGAATGTCC

### Culture of E. coli.

E. coli was routinely cultured in LB broth or agar at 37°C with appropriate antibiotics. Antibiotics used in solid medium were chloramphenicol (10 µg/ml), ampicillin (100 µg/ml), kanamycin (25 µg/ml), and tetracycline (10 µg/ml); antibiotic concentrations were halved for liquid culture.

### Culture of *B. thetaiotaomicron*.

B. thetaiotaomicron was routinely cultured in broth using brain heart infusion medium (BD Biosciences), supplemented with 1.2 µM histidine, 1.9 µM hematin, 1 µg/ml menadione, and 500 µg/ml cysteine (BHI+). B. thetaiotaomicron was cultured in liquid using the pyrogallol method (26): after inoculation, a cotton ball was inserted into the mouth of the culture tube and set aflame; after the flame was extinguished, 200 µl of 20% (wt/vol) NaCO_3_ and 200 µl of 35% (wt/vol) pyrogallol were added to the cotton, and the tube was immediately plugged with a rubber stopper. Cultures of B. thetaiotaomicron were incubated at 37°C, without shaking. Typically, resazurin was added to the liquid medium as an indicator of oxidizing/reducing conditions (1-µg/ml final concentration). B. thetaiotaomicron was cultured on complex medium agar plates using brain heart infusion medium, supplemented with 10% defibrinated horse blood (Bio-Media Unlimited) (BHIH). Minimal medium agar with chondroitin sulfate as the sole carbon source was prepared by dissolving chondroitin sulfate (Sigma-Aldrich or Toronto Research Chemicals) completely in distilled water (for a 5-g/liter final concentration) and autoclaving with agar, followed by adding salts and supplements as previously described (27) as well as trace elements (1,000× stock solution; concentrations per liter: 0.247 g H_3_BO_3_, 0.1 g CuSO_4_⋅5H_2_O, 0.338 g MnSO_4_⋅H_2_O, 0.282 g ZnSO_4_⋅7H_2_O, 0.056 g CoSO_4_⋅7H_2_O, and 0.048 g Na_2_MoO_4_⋅2H_2_O) and the appropriate antibiotics. Antibiotics used in solid medium were gentamicin (200 µg/ml), kanamycin (200 µg/ml), nalidixic acid (25 µg/ml), and erythromycin (10 µg/ml); antibiotic concentrations were halved for liquid culture. Agar plates were incubated in airtight containers with GasPak EZ anaerobe sachets (BD Biosciences).

### Construction of pKL13 fosmid vector.

The fosmid cloning vector pCC1FOS was modified for screening in a B. thetaiotaomicron host. Briefly, the RK2 *oriT* fragment was amplified from pJC8 using primers KL12/KL13 and KOD Hot Start DNA polymerase (Novagen) according to the manufacturer’s recommendations and then digested and cloned into the HindIII site. The *ermF*-*repA* fragment was amplified from pAFD1 using primers KL14/KL15, cloned into the intermediate vector pJET1.2 (Thermo Fisher), and then digested and subcloned into the EcoRI site. The ~4-kb *ermF*-*repA* fragment was sequenced at the Centre for Applied Genomics (Toronto, Canada) to compile the complete sequence for the constructed vector pKL13, using primers KL14, KL16, KL33, KL42, KL43, KL45, and KL46 (Table 2). The vector was further modified to include transcriptional terminators (TTs) that flank the Eco72I cloning site to reduce insert-borne transcription, with both terminators from E. coli MG1655; the TT proximal to *ermF* incorporates the *ilvGEDA* terminator and the TT proximal to the RK2 *oriT* incorporates the *rnpB* T1 terminator (28). Finally, pKL13 contains a stuffer in the Eco72I site that aids in complete digestion of the restriction enzyme site.

### Construction of genomic and metagenomic fosmid libraries.

The metagenomic library was constructed using DNA extracted from a human fecal sample pooled from seven volunteers, obtained with clearance from the Office of Research Ethics of the University of Waterloo. The metagenomic library was designated CLGM3, and the genomic library constructed using genomic DNA from B. thetaiotaomicron VPI 5482 was designated BT3. Library construction was based on methods described previously (29). Briefly, DNA was extracted from either feces or a pure culture of B. thetaiotaomicron VPI 5482 and size selected (~40 to 70 kb) by pulsed-field gel electrophoresis. The insert DNA was electroeluted from the gel fragment, end repaired, and purified for ligation. The fosmid vector pKL13 was prepared by Eco72I digestion followed by dephosphorylation. The insert and vector were ligated, and the ligation products were packaged into lambda phage heads using Gigapack III XL packaging extract (Stratagene). The phage were used to transduce EPI300, and transductants were recovered on LB supplemented with chloramphenicol (10 µg/ml). Colonies were counted to estimate library size and then resuspended, pooled, aliquoted to generate the CLGM3 and BT3 library stocks, and stored at −80°C.

### Triparental conjugation from E. coli to *B. thetaiotaomicron*.

The triparental conjugation protocol was adapted from a biparental protocol (30). Matings were carried out using 5 ml of each of the donor, mobilizer, and recipient strains. The E. coli donor and mobilizer were cultured in 5 ml LB supplemented with the appropriate antibiotics, and the B. thetaiotaomicron recipient was cultured in 5 ml BHI+; all were grown to an optical density at 600 nm (OD_600_) of ~0.4 (Spectronic Spec 20 D). Cells were pelleted by centrifugation at 7,000 × *g* at room temperature for 5 min. The supernatant was removed, and the cells were resuspended in either BHI+ or 1× Bt salts [per liter: 13.6 g KH_2_PO_4_, 0.875 g NaCl, 1.125 g (NH_4_)2SO_4_; pH 7.2 (27)]. Donor, mobilizer, and recipient were mixed in a final volume of 1 ml, and the mixture was swirled evenly over the surface of a BHIH plate. The plate was dried for several minutes in a laminar flow hood and then incubated aerobically overnight with the agar side down. Overnight mating lawns were scraped and resuspended in 2 ml BHI+ or 1× Bt salts. Typically, serial 10-fold dilutions were made from 10^−1^ to 10^−3^, and 100 µl of each dilution was plated on BHIH supplemented with appropriate antibiotics to select for transconjugants; typically, kanamycin or nalidixic acid was used to select against E. coli and erythromycin was used to select for the vector. If the mating was plated on minimal medium, then the mating resuspension was washed to remove complex medium components by three repetitions of centrifugation and resuspension in 1× Bt salts.

### Genomic DNA minipreparation of *B. thetaiotaomicron*.

The minipreparation protocol is based on the method described by Charles and Nester (31). Briefly, B. thetaiotaomicron was cultured in 10 ml of liquid medium with the appropriate antibiotics, and the cell pellets were recovered after centrifugation at 7,000 × *g* for 5 min at room temperature. Cells were resuspended in 400 µl buffer (10 mM Tris [pH 8.0], 25 mM EDTA), 50 µl 5 M NaCl and 10 µl 10-mg/ml RNase A were added, and the tube was inverted several times. Twenty-five microliters of 20% SDS was added, and the sample was incubated at 65°C for 1 to 2 h. Two hundred sixty microliters of 7.5 M ammonium acetate was added, and the sample was incubated on ice for 20 min to precipitate proteins. The mixture was centrifuged at 21,000 × *g* for 20 min, the supernatant was decanted into a new microcentrifuge tube, and the mixture was extracted with chloroform in a 1:1 volume. The DNA was precipitated with 800 µl isopropanol and pelleted by centrifugation at 21,000 × *g* for 5 min. The pellet was washed with 100 µl 70% ethanol and centrifuged at 21,000 × *g* for 1 min, the supernatant was removed, and the pellet was allowed to dry. Finally, the pellet was allowed to dissolve in 50 µl Tris-EDTA (TE) overnight at 4°C, and the genomic DNA was gel quantified against a dilution series of bacteriophage λ DNA (Thermo Fisher) using the software ImageJ (32).

### PCR analysis.

Genomic DNA was isolated from the B. thetaiotaomicron clones carrying *anSME*-complementing fosmid DNA and used as the template. *Taq*-based 2× PCR master mix (Thermo Fisher) was used according to the manufacturer’s recommendations, with the exception that RNase A was added to remove RNA contamination (25-µg/ml final concentration). The touchdown PCR protocol used was 95°C for 3 min; 11 cycles of 95°C for 30 s, 60°C for 30 s (decrease of 1°C per cycle), and 72°C for 1 min/kb; 20 cycles of 95°C for 30 s, 50°C for 30 s, and 72°C for 1 min/kb; and 72°C for 5 min. PCR products chosen for Sanger sequencing were gel extracted, dissolved in binding buffer (140 mM 2-[*N*-morpholino]ethanesulfonic acid [MES]-NaOH [pH 7.0], 20 mM EDTA, 5.5 M guanidine isothiocyanate [33]) at 65°C for 5 min, applied to a silica spin column (BioBasic), washed several times (10 mM Tris-HCl [pH 7.5], 80% ethanol), and eluted in TE buffer. Sanger sequencing was completed at the Centre for Applied Genomics (Toronto, Canada).

### Genome sequencing and analysis.

Genome sequence data were generated on an Illumina MiSeq platform using 250-base paired-end sequencing. Sequence data were aligned to the B. thetaiotaomicron VPI 5482 reference genome (NCBI accession no. NC_004663.1 for the chromosome and NC_004703.1 for the plasmid) using Bowtie 2 version 2.2.6 (34). Sequence data were *de novo* assembled using SPAdes version 3.8.0 (35), and functional annotations were obtained for contigs of interest using RAST (36). Regions of similarity between host and clone DNA were identified using Mauve (37). Data analyses were performed in R, including packages *Rsamtools*, *Gviz*, *ape*, and *genoPlotR*.

### Data availability.

Raw sequence data have been deposited at the NCBI Sequence Read Archive under accession numbers SRX3141910 (CLGM3 clone 2) and SRX3141914 (CLGM3 clone 5). Sequence data and other data may be accessed online at http://www.cm2bl.org/~data, including the raw data, alignment files, genome assemblies, and sequences of the *anSME* ORFs and *ermF*-*repA* fragment, as well as ab1 files from Sanger sequencing. The sequence of the constructed B. thetaiotaomicron-compatible vector, designated pKL13, may be found under NCBI accession no. KU746975. The metagenomic library designated CLGM3 may be found at NCBI BioSample accession no. SAMN04505233, and the genomic library constructed using genomic DNA from B. thetaiotaomicron VPI 5482, designated BT3, may be found at NCBI BioSample accession no. SAMN04505228.
